# Data on transcriptome analysis from mesocarp tissue of mango *Mangifera indica* ‘Chokanan’ fruits

**DOI:** 10.1016/j.dib.2022.108160

**Published:** 2022-04-10

**Authors:** Siti Khadijah A. Karim, Mohamad Zai Mirza Zaini, Zamri Zainal

**Affiliations:** a Faculty of Plantation and Agrotechnology, Universiti Teknologi MARA (UiTM) Melaka, Jasin campus, , Merlimau, Melaka 77300, Malaysia; b Faculty of Applied Sciences, Universiti Teknologi MARA (UiTM) Selangor, Shah Alam, Selangor 40450, Malaysia; c Department of Biological Sciences and Biotechnology, Faculty of Science and Technology, Universiti Kebangsaan Malaysia, Malaysia

**Keywords:** Highly expressed gene, Mango, Transcriptome analysis, Fruit development

## Abstract

Mangoes comes in different sizes and consumer are often favour those with bigger, fleshy mango. Many genes are plays an important role in determining the growth, final size and shapes of the mango. To further understand the roles of genes that play roles in fruit development, a de novo transcriptomic analysis was performed at two stages of fruit development; immature and ripening stage, using Illumina HiSeq 4000 platform with 30× sequencing coverage. A total of approximately 128 Gb of clean nucleotides was obtained from 130 Gb of raw nucletides sequenced from four fruit mesocarp of both time points. The raw and clean data were deposited into National Center for Biotechnology Information (NCBI) Sequence Read Archive (SRA) database with accession number PRJNA803945.

## Specifications Table

**Table:** 

Subject area	Agricultural
Specific subject area	Plant Science
Type of data	RNA sequence data
How data was acquired	Paired end transcriptome of *Mangifera indica* ‘Chokanan’ was sequenced using Illumina HiSeq 4000 sequencing platform. *De novo* transcriptome assembly was performed by using Trinity RNA-Seq v2.4.0.
Data format	Raw reads (FASTQ)
Description of data collection	The mesocarp of M. indica ‘Chokanan’ fruits were obtained from two time points and quickly frozen in liquid nitrogen before stored in −80 °C freezer.
Data source location	City/town/region: Klang, Selangor Country: Malaysia Latitude and longitude (and GPS coordinates) for collected samples/data: 3.008761″N 101.489859″E
Data accessibility	The raw FASTQ files of immature and ripen ‘Chokanan’ were deposited at National Center for Biotechnology Information (NCBI) Sequence Read Archive (SRA) (https://www.ncbi.nlm.nih.gov/sra) with BioProject accession number PRJNA803945 and the accession number for ripen SAMN25690807 (https://www.ncbi.nlm.nih.gov/sra/?term=SAMN25690807) and immature SAMN25690806 (https://www.ncbi.nlm.nih.gov/sra/?term=SAMN25690806)

## Value of the Data

• The data obtained using Illumina sequencer is the first source of M. indica ‘Chokanan’ RNA-seq.
• The data provide a glimpse into molecular perspectives of M. indica ‘Chokanan’ and contribute further to gene manipulation for interest experiment.
• The data presented here can be used for fruit quality improvement in mango varieties through the identification of molecular markers such as single nucleotide polymorphisms (SNPs) and microsatellites.
• The genetic information and gene sequences can contribute to transcriptomic database.

## 1 Data

The RNA of a Malaysian mango cultivar ‘Chokanan’ was used for de novo transcriptome analysis using Illumina HiSeq 4000 sequencing technology, with the read length of 150 bp at each end. We used a 30× depth of sequencing coverage. Approximately 130 Gb raw data sequenced were generated from four samples of two stages Chokanan mango fruits. Table 1 shows the summary of raw and clean reads that have been generated from the transcriptomic sequencing of immature and ripen fruits of mango ‘Chokanan’ variety. The clean raw reads have been deposited at National Center for Biotechnology Information (NCBI) Sequence Read Archive (SRA) (https://www.ncbi.nlm.nih.gov/sra) under the BioProject accession number PRJNA803945.

**Table 1:** Summary of RNA-seq generated from two different libraries.

Growth stage	Fruit phenotype	Raw reads (bp)	Clean reads (bp)	SRA accession number
Immature (21 DAP)	Small, green	65219966	63992745	SAMN25690806
Ripening (75 DAP)	Large, yellow	65526463	64598345	SAMN25690807
Total Nucleotides		130746429	128591090	

## 2 Experimental Design, Materials and Methods

### 2.1 Fruits harvest

Mango fruits were harvested at two time points corresponding to immature and ripening stages within the same season. Fruits were harvested from two trees that were grown on the same soil and from the same rootstock, thus minimizing any external factor and genetic variation that may affects the fruit development. The fruits were harvested according to the Days After Pollination (DAP) which was on 21st DAP for immature stage, and at 75th DAP for the ripening stage [1]. Three fruits were harvested from each tree for each time points. Two of the fruits were used for RNA extraction which was needed for the transcriptomic analysis purpose. The fruits were dissected by removing the skin where the mesocarp was cut into 1 cm cubes. The mesocarp was quickly frozen in liquid nitrogen and stored in −80 °C until further use.

### 2.2 Total RNA isolation and cDNA synthesis

Two biological replicate of mango fruits mesocarp from each stages were sent to the laboratory of Apical Scientific Sdn Bhd (Malaysia) for RNA isolation and cDNA synthesis. Transcriptome data were generated from the total RNA extracted from these two samples collected at two different developmental stages. This resulting in four RNA-Seq for further downstream analysis. NanoDrop spectrophotometer (Thermo Scientific, Waltham, MA, USA) and Bioanalyzer RNA 6000 chips system (Agilent Technologies, USA) were used to determine the quality and integrity of total RNA. The 0.8% agarose gel was used to examine the integrity of RNA samples.

### 2.3 Transcriptomic sequencing

The paired-end transcriptomic sequencing and analysis were carried out by Apical Scientific Sdn Bhd (Malaysia) using Illumina HiSeq 4000 sequencing platform with the read length of 150 bp at each end. Paired-end sequencing libraries with insert sizes of 350 bp were constructed prior to sequencing following the manufacturer's standard protocol (Illumina, San Diego, CA, USA). The raw reads generated from the samples were trimmed using Solexa QA++ with Phred score Q20. By using FastQ file, the FastQC was ran at default parameter. De novo assembly of the data was done using Trinity RNA-Seq 2.0 with default settings [2].

## CRediT authorship contribution statement

**Siti Khadijah A. Karim:** Conceptualization, Methodology, Supervision, Software, Validation, Writing – review & editing. **Mohamad Zai Mirza Zaini:** Formal analysis, Resources, Writing – review & editing. **Zamri Zainal:** Conceptualization, Writing – review & editing.

## Declaration of Competing Interest

The authors declare that they have no known competing financial interests or personal relationships that could have appeared to influence the work reported in this paper.

## Data Availability

Data on transcriptome analysis from mesocarp tissue of mango Mangifera indica ‘Chokanan’ immature fruits (Original data) (NCBI). Data on transcriptome analysis from mesocarp tissue of mango Mangifera indica ‘Chokanan’ ripe fruits (Original data) (NCBI).

## References

[1] Prasanna V., Prabha T.N., Tharanathan R.N. (2007). Fruit ripening phenomena: an overview. Crit. Rev. Food Sci. Nutr..

[2] Grabherr M.G., Haas B.J., Yassour M., Levin J.Z., Thompson D.A., Amit I., Adiconis X., Fan L., Raychowdhury R., Zeng Q., Chen Z. (2011). Full-length transcriptome assembly from RNA-Seq data without a reference genome. Nat. Biotechnol..

